# Placebo, nocebo: Believing in the field of medicine

**DOI:** 10.3389/fpain.2022.972169

**Published:** 2022-07-29

**Authors:** Karin Meissner

**Affiliations:** ^1^Division of Health Promotion, Coburg University of Applied Sciences and Arts, Coburg, Germany; ^2^Medical Faculty, Institute of Medical Psychology, Ludwig-Maximilians-University Munich, Munich, Germany

**Keywords:** placebo effect, nocebo effect, belief, expectation, hope, prediction error

## Introduction

A medical treatment is regarded efficacious if it induces a larger improvement than an inert placebo treatment. The efficacy of the active treatment is usually tested in randomized placebo-controlled trials, which are expensive but necessary because also placebo treatments are associated with large improvements. This improvement is due in part to the occurrence of a “placebo effect.”

The placebo effect is a genuine phenomenon that has been intensively researched in recent decades. A placebo treatment is by definition an inert treatment without specific ingredients, for example, a pill without pharmacologic ingredients. The placebo effect is best conceptualized as the effect of the informational context in which a (placebo or active) treatment is embedded and which consists of internal and external cues (1). External cues comprise, for example, the care provider's verbal suggestions about the effects of a treatment (e.g., “this drug is a powerful painkiller”) as well as associated non-verbal cues conveyed through body language and facial expression. External cues include also the characteristics of a particular treatment, such as its invasiveness, price, color, and the medical setting in which it is applied (2). Internal cues that play a role in the formation of placebo effects comprise pre-existing expectancies, previous experience and the affective state of a patient. Internal and external cues interact with each other, and the resulting informational context can be considered the “active ingredient” of placebo interventions (1).

Typically, the informational treatment context translates into specific treatment expectations. Positive treatment expectations are thought to trigger placebo effects, that is, beneficial effects on health-related outcomes. When negative treatment expectations arise, so-called “nocebo effects” can occur, resulting, for example, in the occurrence or aggravation of symptoms. Treatment expectations are also able to modulate the effects of active treatments (3, 4). Negative treatment expectations are typically elicited by information about the risks of a treatment, communicated through healthcare professionals, medication leaflets, mass media, social media, and other patients (5). Nocebo effects have often been studied by analyzing the adverse events in the placebo arms in clinical trials. For example, a recent meta-analysis on the side effects of COVID-19 vaccination found that 76% of the systemic side effects after the first dose of vaccine, such as headache and fatigue, were also seen in the placebo groups, suggesting that the majority of systemic side effects were due to nocebo effects (6).

Placebo and nocebo effects can affect almost any medical symptom, including but not limited to pain, itch, nausea, depression, and motor symptoms (7). Notably, also physiological parameters, such as autonomic activity (8, 9) and plasma proteins (10) have been shown to be affected by placebo interventions. Furthermore, placebo effects can be surprisingly system specific: According to the content of the accompanying verbal suggestion, placebo interventions specifically affected gastric activity but not cardiovascular activity (11), and blood pressure but not gastric activity (12).

## Neurobiological mechanisms

The neurobiological mechanisms underlying placebo effects differ depending on the conditions and paradigms used to induce placebo effects. According to their diversity, different neurochemical systems are known to be involved, including the opioid, dopamine, cholecystokinin, and oxytocin systems (1). For example, the opioid antagonist naloxone partially blocks placebo analgesia, whereas the cholecystokinin-antagonist proglumide inhibits the nocebo hyperalgesia, suggesting the involvement of opioidergic and cholecystokininergic pathways (13, 14). A recent meta-analysis of individual patient data from fMRI studies focusing on pain provided strong evidence for placebo-associated reductions of pain-related activity in areas linked to nociception and pain, such as the insular and thalamic regions. These changes, in turn, correlated with the magnitude of behavioral pain reduction (15). Effect sizes, however, were small, suggesting that further mechanisms underly placebo effects in pain. The meta-analysis also revealed increased activity in front-oparietal brain regions during placebo analgesia, particularly in the dorsolateral prefrontal cortex (DLPFC). This activation is thought to mirror the construction of top-down representations of context, including expectations and beliefs (15). The pivotal role of the DLPFC for placebo effects is nicely illustrated by an experimental study showing that the disruption of the DLPFC by repetitive transcranial magnetic stimulation completely blocked the placebo analgesic effect (16). In addition, the meta-analysis of placebo brain imaging studies showed a reduction of activity in brain areas related to negative affect (15). Accordingly, experimental evidence suggests that placebo effects on pain are partly mediated by reduced negative affect (17, 18), possibly induced by cognitive re-appraisal strategies (15). A further brain region frequently activated during placebo hypoanalgesia is the ventromedial prefrontal cortex (vmPFC), an area with a prominent role in decision making, valuation, and choice (1). VmPFC activation during placebo analgesia may reflect the occurrence of a decision bias evoked by the brain in an ambiguous situation. When positive treatment expectations, for example, let expect less pain while the nociceptive stimulus actually remains the same, a prediction error occurs. The brain may resolve this prediction error by a placebo hypoalgesic effect (19).

The majority of placebo effects are likely to be due to emotional re-appraisal strategies and cognitive-evaluative processes. Only very strong placebo interventions, such as induced by classical conditioning or powerful manipulations of belief, may affect early sensory processes in a significant manner (1).

## Social neuropeptides and placebo effects

Allo-grooming in animals signals intense social relationships, and it has been postulated to constitute an important evolutionary trace of the placebo effect in humans (20–22). Indeed, empathetic behavior can enhance placebo effects. In a randomized controlled clinical trial on irritable bowel syndrome, for example, sham acupuncture was administered by a healthcare provider who was either instructed not to talk to the patients, or to interact with patients in an empathetic manner. Addition of empathy further enhanced the magnitude of the placebo effect induced by sham acupuncture (23). Furthermore, there is experimental evidence that neuropeptides released during social interactions, including oxytocin and vasopressin, can modulate placebo hypoalgesia (24, 25). For example, Colloca et al. (25) performed a randomized, placebo-controlled trial, in which nasal vasopressin agonists were administered to healthy volunteers before placebo analgesia was induced. The results showed that vasopressin remarkably enhanced the analgesic effect of the placebo intervention in women. By using plasma proteomics, we recently provided first evidence that the neuropeptides neurexin 1 (NRXN1), contactin-associated protein-like 4 (CNTNAP4), and reelin (RELN) play a role for the placebo effect in nausea (10). The cell adhesion molecules NRXN1 and CNTNAP4 are involved in mirror neuron activity and empathic behavior and have been linked to grooming behavior, and RELN is known to functionally interact with oxytocin. These preliminary results of an unbiased methodological approach (i.e., without a priori hypotheses) are promising, as they confirm previous findings that trust and a good doctor-patient relationship can improve medical outcomes and that such effects have a biological basis.

## Open-label placebos

One of the most spectacular results of recent placebo research was the discovery that the open-label administration of placebos, where the patient is truthfully informed that the pill contains no pharmacological substance, produces a placebo effect. Since the first pilot study in patients with irritable bowel syndrome (26), numerous trials have confirmed that open-label placebos can positively affect a variety of medical conditions, including but not limited to chronic low back pain, chronic knee pain, episodic migraine, allergic rhinitis, depression, attention deficit hyperactivity disorder, and cancer-related fatigue (27–32). There is even evidence that the beneficial effects of open-label placebos can last for several years (33).

The mechanisms underlying the effects of open-label placebos are largely unknown. A qualitative study in patients receiving open-label placebo within a clinical trial suggested that hope, rather than expectation, may play a role (19). While expectations refer to a relatively high (assumed) likelihood of the desired outcome and represent a rather cognitive construct, hope can also be present when the likelihood is very low and has often been conceptualized as an emotional state (34). Hope can drive patients to seek treatment even from a counterintuitive intervention such as open-label placebos (19).

Kaptchuk et al. (19) suggested that prediction error processing could explain the hypoalgesic effects of both deceptive and open-label placebos: In the case of deceptive placebo administration, positive expectations primarily lower the level of predicted pain, resulting in a prediction error which is resolved by the brain through a perceived hypoalgesic effect. In the case of OLP treatment, the placebo effect could be primarily due to reduced precision of the predicted pain signal, i.e., increased uncertainty resulting from the paradox information of receiving “substances that have no active ingredients.” According to Bayesian brain models, the lowered precision of the “prior” (i.e., predicted pain) also leads to a prediction error, which in turn is resolved by a perceived hypoalgesic effect (19). Previous research on open-label placebos thus suggests that placebo effects can be elicited also in the absence of expectations, for example, when the patient is in an affective state of hope and increased uncertainty. Bayesian brain models provide a comprehensive model to explain both types of placebo effects.

## The temporal dynamics of placebo effects

The multitude of mechanisms involved in placebo effects shows that this neurobiological phenomenon is complex and multifaceted. The temporal dynamics of placebo effects, however, have rarely been studied. Several authors suggested that perceived active treatment assignment may increase expectations, and thus placebo effects over time (35–37). In a randomized controlled trial in depression, for example, perceived treatment assignment affected symptom improvement only in the second half of the trial (37). “Active” placebo interventions that deliver non-specific sensory stimuli may be particularly useful in initiating such reinforcement processes. For example, adding electrotactile stimulation to a sham electrical nerve stimulation intervention for nausea significantly increased study participants' belief that they had received the “active” intervention. Although the magnitude of the placebo effect at the first placebo administration did not differ between the two placebo conditions, the difference in perceived treatment assignment could well lead to higher treatment expectations and thus placebo effects at subsequent placebo administrations (36). The long-lasting improvements in chronic low back pain observed during the 5-year follow-up of an open-label placebo study (33) furthermore suggests that placebo interventions can trigger strong and salient changes in patients' belief systems that may have long-term health effects. Altered cognitions, emotions and re-appraisal strategies, as well as changes in health behavior, may mediate such long-lasting placebo effects. Finally, also nocebo effects are most likely subject to changes over time, although empirical evidence in this area is limited due to ethical constraints.

## Placebo effects and the process of believing

As outlined above, placebo research indicates that treatment expectations and related beliefs are not stable but are subject to change. Recently, a new area of research has emerged that aims to better understand beliefs as a function of “credition,” that is, the “process of believing” (derived from the Latin verb “credere” - “to believe”) (38). The process of believing is conceptualized as a basic brain function with neurophysiological underpinnings (39) that links past experience with predictions about the future and enables individuals to make sense of signals in the environment and ascribe personal meaning to them (38). Beliefs are the neural representations that result from the ongoing process of believing and can be reinforced and updated through learning processes. The model of credition thus shares many similarities with recent concepts in placebo research and offers a promising approach to better understand the dynamic formation of treatment-related beliefs and expectations as well as their clinical effects.

## Concluding remarks

Placebo effects are not unique responses, but comprise a variety of mechanisms that differ between conditions and research paradigms. They rely on the brain's ability to actively integrate contextual information with prior experiences, conceptual knowledge, beliefs, and emotions, resulting in brain responses that promote health and well-being (1). There is considerable overlap with emerging concepts such as predictive coding and the process of believing. Integrating these concepts into placebo research could provide a better understanding of the fluid nature of beliefs and expectations and their role in maintaining health and combating disease.

## Author contributions

The author confirms being the sole contributor of this work and has approved it for publication.

## Funding

This paper was funded by Rüdiger Seitz, *via* the Volkswagen Foundation, Siemens Healthineers, and the Betz Foundation. The funders were not involved in the study design, collection, analysis, interpretation of data, the writing of this article or the decision to submit it for publication.

## Conflict of interest

The author declares that the research was conducted in the absence of any commercial or financial relationships that could be construed as a potential conflict of interest.

## Publisher's note

All claims expressed in this article are solely those of the authors and do not necessarily represent those of their affiliated organizations, or those of the publisher, the editors and the reviewers. Any product that may be evaluated in this article, or claim that may be made by its manufacturer, is not guaranteed or endorsed by the publisher.
